# Problem gambling in adolescents: what are the psychological, social and financial consequences?

**DOI:** 10.1186/s12888-019-2293-2

**Published:** 2019-10-22

**Authors:** Goran Livazović, Karlo Bojčić

**Affiliations:** Department for Pedagogy, Faculty of Humanities and Social Sciences, University of J. J. Strossmayer in Osijek, Lorenz Jaeger Street 9, 31000 Osijek, Croatia

**Keywords:** Gambling, Family, School, Adolescents, Risk behaviour, Sociodemographic traits

## Abstract

**Background:**

The paper examines the roles of sociodemographic traits, family quality and risk behaviour in adolescent problem gambling, with focus on the psychological, social and financial consequences from the socio-ecological model approach. This model emphasizes the most important risk-protective factors in the development and maintenance of problem gambling on an individual level, a relationship level, as well as a community and societal level.

**Methods:**

The research was done using the *Canadian Adolescent Gambling Inventory* with a sample of 366 participants, 239 females (65.3%) using descriptive statistics and t-test, ANOVA, correlation and hierarchical regression analysis.

**Results:**

Males reported significantly higher gambling consequences on all scales (*p* < .001) and significantly more risk behaviour (*p* < .05). Age was significant for psychological consequences (*p* < .01), problem gambling (*p* < .01) and risk behaviour (*p* < .001) with older participants scoring higher. Students with lower school success reported significantly higher psychological consequences of gambling (*p* < .01), higher risk behaviour activity (*p* < .001) and lower family life satisfaction (*p* < .001). The psychological, financial and social consequences were positively correlated with problem gambling (*p* < .001). Age (*p* < .05), gender (*p* < .001), school success (*p* < .01) and the father’s education level (*p*. < 05) were significant predictors of problem gambling, with older male adolescents who struggle academically and have lower educated fathers being at greater risk.

**Conclusions:**

Results indicate an important relation between adolescent gambling behaviour and very serious psychological, social and financial consequences. There is a constellation of risk factors that likely place certain individuals at high risk for problem gambling.

## Background

In this article, we present a socio-ecological analysis of significant sociodemographic, family, school and gambling related factors predicting problem gambling among adolescents, as well as the most important empirical conclusions based on survey results with 366 participants, with special focus on the role of psychological, social and financial consequences, as well as risk-protective factors related to sociodemographic traits, family, school and adolescent risk behaviour. The *socio-ecological model* includes risk and protective factors on an *individual level* (health and personal traits), a *relationship level* (the closest social circle that contributes to the range of experience); the *community level* (the settings for interaction); and the *societal level* (social and cultural norms, as well as diverse social policies) [1]. The gambling panorama has shifted significantly during the past decades, from an initially mild type of entertainment to a hazardous addiction resulting in a number of academic, behavioural, personality, social, interpersonal, financial, criminal or mental health difficulties for children and adolescents experiencing gambling-related problems [2–4]. Current frameworks conceptualise problem gambling across a risk continuum [5], as the term describes gambling behaviour that results in adverse consequences for individuals, families and communities [6]. These consequences can range from impaired mental health, physical health, relationship and family dysfunction, to financial problems, employment difficulties and legal issues [7].

### Research on the characteristics and risk-protective factors in gambling

Despite being illegal for minors, gambling is a common activity among adolescents. On an international level, estimates of past year gambling participation and problem gambling in youth (from 2000 to 2009) were highly variable, with rates of 0.8 to 6.0% [8], suggesting they exceed those of adults [9–12]. Researchers have reported evidence that a combination of biological, psychological and social factors contribute to gambling behaviour [13, 14]. A recent meta-analyses by Dowling et al. emphasized 13 individual risk factors (alcohol use frequency, antisocial behaviours, depression, male gender, cannabis use, illicit drug use, impulsivity, number of gambling activities, problem gambling severity, sensation seeking, tobacco use, violence, under-controlled temperament), one relationship risk factor (peer antisocial behaviours), one community risk factor (poor academic performance), one individual protective factor (socio-economic status) and two relationship protective factors (parent supervision, social problems) [12]. A number of problem gambling cross-sectional studies identified female gender, adaptive coping strategies, emotional intelligence, well-being, self-monitoring, personal competence, resilience, interpersonal skills, social competence, social support and bonding, school connectedness, parental monitoring and family cohesion as protective factors [3, 12, 15–22]. Integrated models of pathological gambling such as the *Pathways model*, introduced by Nower and Blaszczynski, suggest that a number of biological, personality, developmental, cognitive and environmental factors can be incorporated into a theoretical framework that helps explain youth gambling behaviour [23]. Derevensky et al. and Gupta and Derevensky suggest that in addition to helping youth understand the laws of probability, as well as independence of events and erroneous cognitions, attention must be paid to the underlying motivations leading to excessive adolescent pathological gambling (e.g., depressive symptomatology; somatic disorders; anxiety; attention deficits; self-regulation difficulties; academic, personal and familial problems; mood disorders; high risk-taking or poor coping skills) [24, 25]. Researchers also emphasize risk factors such as parental/familial gambling and parental/familial approval of gambling, antisocial behaviour, deviant peers, substance abuse and school problems [14, 26–30]. Problem gambling comorbid with mental health disorders has been associated with increased psychiatric symptoms; substance use problem severity; interpersonal, physical, financial and social difficulties; impulsivity and suicidality [31]. These factors play a role in the development and/or maintenance of gambling behaviour and problem gambling [27]. On the other hand, family life satisfaction (cohesion) and the quality of school affiliation, as indicators of social bonding, have been identified as protective factors in relation to youth gambling problems [13, 14, 30].

### The present study

This research was aimed on explicating the role of sociodemographic traits (age, gender, residence in urban or rural surroundings) in profiling and predicting the average problem gambler characteristics, as well as studying the impact of traditional protective factors such as family life quality (lower/higher personal satisfaction), family structure (both parents/ other), the parents’ educational (lower/higher education) and professional status (employed/unemployed) and school related factors (school type, academic success). Special emphasis was placed on the relation between problem gambling behaviour and the psychological / social / financial consequences, as well as adolescent risk behaviour (physical violence/ alcohol/ drugs/ smoking/ risky sexual behaviour/school truancy/ deliberate destruction of property) from a predictive perspective. Based on the research aim and problems, the following hypotheses were established: (H1) sociodemographic traits have a significant impact on adolescent gambling; (H2) family life satisfaction, parental traits and school related factors represent protective factors in gambling aetiology; and (H3) problem gambling has significant psychological, social and financial consequences on adolescents.

## Methods

### Study design and research goal

The aim of the study was to examine the role of sociodemographic traits, family relations and parent characteristics, as well as school related factors in adolescent gambling behaviour with special focus on its psychological, social and financial consequences.

### Statistical analysis

Quantitative analyses were conducted using descriptive and inferential procedures. The data was first processed for central tendency values on all measured items. The results were obtained using the t-test for independent samples and ANOVA concerning gender, age, place of residence, family structure, school success, school type, parents’ educational level and the parents’ employment status. A correlation analysis was implemented as to investigate the relation between sociodemographic traits, family life quality, risk behaviour, problem gambling and its psychological, social and financial consequences. A hierarchical regression analysis was implemented as to establish the most significant predictors for adolescent problem gambling. Due to a limited sample size and distribution, in order to perform the inferential tests, the “Age” variable was recoded into 2 groups, younger (14, 15 and 16) and older adolescents (ages 17–20). The variable “School success” was recoded into 3 groups, lower achievement (grades 1, 2 and 3 or F, D, C), average achievement (grade 4 or B) and higher achievement (grade 5 or A). The variable “Parent employment” was recoded into 2 groups, the employed and unemployed/other. The variable “Father’s education level” and “Mother’s education level” were both recoded into 2 groups, lower educated and higher educated fathers/mothers. The data were tested using the Shapiro-Wilk normality test for *age* (*W = .821; p=,000*) and *gender* (*W = .602; p=,000*).

### Measures and data collection

A multidimensional questionnaire was constructed for the purpose of this research. The five-degree pen-paper Likert-scale survey consisted of 4 parts in Croatian language.

a) The first part encompassed questions about sociodemographic traits (gender, age, school type, parent education level, family economic well-being, urban or rural residence, academic success, study programme).

b) The second part consisted of a non-standardised 4 item scale with questions on family life quality (i.e. *I get along with my parents; I can ask my parents for help; My family agrees on rules mutually*), that was computed and transformed into a new composite variable named “Family life quality”, with consequent reliability analysis showing a high Cronbach’s alpha coefficient (α = .97).

c) The third part consisted of a non-standardised 8 item scale with questions on risk behaviours, which was computed and transformed into a new composite variable named “Risk behaviour” (i.e. I use physical violence to solve problems; I smoke cigarettes; I drink alcohol; I deliberately destroy property…), with consequent reliability analysis showing a satisfactory Cronbach’s alpha coefficient (α = .70).

d) The last, fourth part of the questionnaire was based on the Canadian Adolescent Gambling Inventory [32, 33], with 3 standardised subscales on the financial consequences, the social consequences and the psychological consequences scale. The CAGI was developed specifically for adolescents [34, 35]. It is a paper-and-pen survey with 44-items, aimed at measuring the range and the complexity of gambling behaviour, rather than a dichotomy of either presence or absence of problem gambling, as is found in most existing adolescent and adult instruments [32, 33]. The CAGI has 19 items that measure gambling frequency using six-point response options and time spent gambling in a typical week on 19 forms of gambling and two items to measure money and items of value lost gambling [33]. It measures four gambling-related domains of loss of control, social, psychological and financial consequences, and a fifth, Gambling Problem Severity Scale (GPSS). In our study, the social consequences scale (i.e. *I missed sports practice or other activity because of gambling; I missed a family gathering because of gambling…*) had a satisfactory Cronbach’s alpha coefficient (α = .67), the psychological consequences scale (i.e. *I felt guilty for losing money on gambling; I felt sad or depressed because of the amount of money I lost gambling*) had a high Cronbach’s alpha coefficient (α = .93), and the financial consequences scale (i.e. *I borrowed money for gambling; I stole to obtain money for gambling or repaying debts…*) had a high Cronbach’s alpha coefficient (α = .85), while the Gambling Problem Severity Scale (GPSS), (i.e. *I planned gambling; After gambling, I returned to try win back the lost money…*) also had a high Cronbach’s alpha coefficient (α = .88). In previous research studies, CAGI was found to yield satisfactory estimates of reliability, validity and classification accuracy [32, 33]. Studies have shown it is an appropriate instrument for epidemiological studies as well as for clinical and school settings [32–35].

### Participants

A convenience sample was recruited to reflect the characteristics of the adolescent population. The research was conducted with 366 participants, 239 female and 127 male adolescents aged *14* (*N* = 35; 9.6%), *15* (*N* = 142; 38.8%), *16* (*N* = 20; 5.5%), *17* (*N* = 25; 6.8%), *18* (*N* = 133; 36.3%), *19* (*N* = 9; 2.5%) and *20* (*N* = 2; 0.5%). A total of 98 (37.8%) participants reported living in rural areas, and 161 (62.2%) lived in urban surroundings. 144 (39.3%) participants attended gymnasiums, and 222 (60.7%) vocational schools. 292 (79.8%) reported living with both parents, 13 (3.6%) only with their fathers, and 53 (14.5%) with their mothers, while 8 (2.2%) live with someone else. The 366 participants had a 4.1 GPA with 32.2% (*N* = 118) achieving an A (excellent), 53% (*N* = 194) achieving a B (very good), 12.6% (*N* = 46) achieving a C (good) and 2.2% (*N* = 8) that failed class (F). Participants reported that 6% (*N* = 26) of their fathers completed only elementary school, 229 (62.6%) finished high school, 47 (12.8%) completed college and 63 (17.3%) had university education. Some 6% (*N* = 23) of mothers completed elementary school, 225 (61.5%) finished high school, 42 (11.5%) completed college and 76 (20.8%) had university education.

### Ethics and study procedure

A paper-pen survey with high-school participants was conducted in March 2018 via group assessment during class in the Osječko-baranjska and Vukovarsko-srijemska region in Croatia. Participants were introduced to the research goal prior to responding and given instructions on the procedure, as well as basic definitions on gambling behaviour. Individuals were excluded if they were unable to understand and provide informed consent. All participants were informed and guaranteed complete anonymity, in line with the Ethical Code of Research with Children [36]. Procedures performed in studies involving human participants were in accordance with the ethical standards of the institutional and/or national research committee, and with the 1964 Helsinki declaration and its later amendments or comparable ethical standards. A trained research assistant was responsible for the survey implementation. The research assistant was present at all times during the survey procedure and helped with possible explanations and survey guidelines. After completion, the participants were asked to check the survey answers as to guarantee responding to all the questions. The data was processed using SPSS (v20.0.0) with descriptive and inferential statistical analysis, t-test and one-way ANOVA, correlation analysis and hierarchical regression analysis.

## Results

### Descriptive and inferential analysis

#### Sociodemographic characteristics and gambling

The earliest reported age of gambling initiation was 7 (*N* = 1, 0.3%), while gambling initiation was most prevalent at ages 14 (*N* = 13, 3.6%), 15 (*N* = 10, 2.7%) and 18 (*N* = 11, 3%). Out of 366 participants, 154 (42.1%) participants reported never having gambled. Our results show that playing cards, dares and challenges or skills represent the most prevalent adolescent gambling activities (Table 3), while 6.6% (*N* = 24) of them bet regularly in sports betting houses and 5.8% (*N* = 21) do it online, 3% (*N* = 11) regularly bet on casino slot machines and 3.6% (*N* = 13) bet on live casino games, and 6.9% (*N* = 25) bet on casino games online (Table 1).

**Table 1:** Descriptive analysis on adolescent gambling preferences

Variable	Group		never	rarely	sometimes	often	always	∑
Playing cards	Male	N	62	17	20	12	16	127
		%	16.9	4.6	5.5	3.3	4.4	34.7
	Female	N	179	28	18	8	6	239
		%	48.9	7.7	4.9	2.2	1.6	65.3
∑		N	241	45	38	20	22	366
		%	65.8	12.3	10.4	5.5	6	100
Sports betting house	Male	N	79	9	18	10	11	127
		%	21.6	2.5	4.9	2.7	3	34.7
	Female	N	225	8	3	3	0	239
		%	61.5	2.2	0.8	0.8	0	65.3
∑		N	304	17	21	13	11	366
		%	83.1	4.6	5.7	3.6	3.0	100
Slot machines at casinos	Male	N	96	11	12	4	4	127
		%	26.2	3	3.3	1.1	1.1	34.7
	Female	N	234	1	1	3	0	239
		%	63.9	0.3	0.3	0.8	0	65.3
∑		N	330	12	13	7	4	366
		%	90.2	3.3	3.6	1.9	1.1	100
Live games at casinos	Male	N	99	9	8	6	5	127
		%	27	2.5	2.2	1.6	1.4	34.7
	Female	N	232	3	2	2	0	239
		%	63.4	0.8	0.5	0.5	0	65.3
∑		N	331	12	10	8	5	366
		%	90.4	3.3	2.7	2.2	1.4	100
Internet sports betting house	Male	N	93	9	7	6	12	127
		%	25.4	2.5	1.9	1.6	3.3	34.7
	Female	N	229	6	1	3	0	239
		%	62.6	1.6	0.3	0.8	0	65.3
∑		N	322	15	8	9	12	366
		%	88	4.1	2.2	2.5	3.3	100
Internet casino games	Male	N	84	11	14	10	8	127
		%	23	3	3.8	2.7	2.2	34.7
	Female	N	206	17	9	6	1	239
		%	56.3	4.6	2.5	1.6	0.3	65.3
∑		N	290	28	23	16	9	366
		%	79.2	7.7	6.3	4.4	2.5	100
Own or someone else’s performance in games of skill	Male	N	47	29	24	19	8	127
		%	12.8	7.9	6.6	5.2	2.2	34.7
	Female	N	141	50	23	17	8	239
		%	38.5	13.7	6.3	4.6	2.2	65.3
∑		N	188	79	47	36	16	366
		%	51.4	21.6	12.8	9.8	4.4	100
A dare or challenge that you or someone else can do something	Male	N	58	22	27	12	8	127
		%	15.8	6	7.4	3.3	2.2	34.7
	Female	N	162	34	22	15	6	239
		%	44.3	9.3	6	4.1	1.6	65.3
∑		N	220	56	49	27	14	366
		%	60.1	15.3	13.4	7.4	3.8	100
Video games for money or something of value	Male	N	87	15	11	9	5	127
		%	23.8	4.1	3.0	2.5	1.4	34.7
	Female	N	218	12	4	1	4	239
		%	59.6	3.3	1.1	0.3	1.1	65.3
∑		N	305	27	15	10	9	366
		%	83.3	7.4	4.1	2.7	2.5	100

Our t-test for gender differences results show that *gender* is a significant factor for psychological (*p* < .001), social (*p* < .001) and financial consequences (*p* < .001) of gambling, as well as problem gambling (*p* < .001) and risk behaviour (*p* < .05), with males reporting significantly higher values on all scores (Table 2).

**Table 2:** T-test for gender differences

Variable	Gender	N	Mean	SD	t
Psychological consequences of gambling	M	127	6.48	3.76	7.18***
	F	239	4.44	1.68	
Social consequences of gambling	M	127	4.72	1.69	4.18***
	F	239	4.16	.89	
Financial consequences of gambling	M	127	4.42	1.29	3.89***
	F	239	4.06	.45	
Family relations quality	M	127	7.35	6.45	.32
	F	239	7.13	6.26	
Problem gambling	M	127	10.64	4.19	7.03***
	F	239	8.47	1.66	
Risk behaviour	M	127	11.61	4.74	2.61*
	F	239	10.44	3.70	

Note: *p < .05; **p < .01; ***p < .001

Our results were not significant for the social and financial consequences of gambling in relation to *age*, but showed significant *age* differences in psychological consequences (*p* < .01), problem gambling behaviour (*p* < .01) and risk behaviour (*p* < .001) with older participants scoring higher (Table 3).

Our research has shown (Table 4) that lower school achievers report significantly higher psychological consequences of gambling in relation to more successful students (*p* < .01).

Our results show no significant differences in gambling behaviour or its consequences in relation to the school type participants attend (Table 5), but indicate significantly higher risk behaviour for vocational school students (*p* < .001).

##### Results on family and parent characteristics in gambling aetiology

The place of residence (urban/rural) was significant for the quality of family relations t(362) = 10.03; *p* < .001, with rural participants reporting significantly higher family life satisfaction (*N* = 98; M = 12.07), but more risk behaviour as well, t(362) = 2.26, *p* < .05. Family structure (both parents/other) was significant for family life quality t(362) = − 2.35, *p* < .05, with participants from structurally deficient families reporting higher family life satisfaction (*N* = 74, M = 8.74), and for risk behaviour t(362) = − 2.50, *p* < .01, as participants from structurally deficient families reported more risky behaviour (*N* = 74, M = 11.90). Our results show the mothers’ employment status was significant only for family life satisfaction, as those who had employed mothers reported more satisfaction t(362) = 3.71, *p* < .001 (*N* = 245, M = 8.01). The fathers’ employment status was not significant for any of the examined variables. Both the mothers and fathers’ educational level did not show significance in relation to any of the examined variables.

### Correlation and regression analysis results for problem gambling

Our results (Table 6) show a moderate to strong positive correlation between the psychological, financial and social consequences with problem gambling (*p* < .001). There was a positive moderate correlation between problem gambling and risk behaviour, as well (*p* < .001). Participants reported a positive weak correlation between school success and the psychological (*p* < .001) and social consequences of problem gambling (*p* < .01).

Our hierarchical regression analysis results show that age (*p* < .05), gender (*p* < .001), school success (*p* < .01) and the father’s education level (*p*. < 05) are significant sociodemographic predictors of problem gambling in adolescents, with older male adolescents who struggle academically and have lower educated fathers being at greater risk (Table 7). Interestingly, our results show that more psychological, social and financial consequences in gambling positively predict problem gambling (*p* < .001).

## Discussion

This study was focused on explicating the role of sociodemographic traits (age, gender, residence in urban or rural surroundings) in profiling and predicting the average problem gambler characteristics, as well as studying the impact of traditional protective factors such as family life quality, family structure, the parents’ educational and professional status and school related factors. Special emphasis was placed on the relation between problem gambling behaviour and the psychological / social / financial consequences, as well as adolescent risk behaviour.

### Adolescent gambling preferences

Derevensky and Gilbeau indicate that typical forms of teen gambling include: card playing for money (poker), sports wagering, dice and board games with family and friends; betting with peers on games of personal skill (e.g., pool, bowling, basketball); arcade or video games for money; purchasing lottery tickets; wagering at horse and dog tracks; gambling in bingo halls and card rooms; playing slot machines and table games in casinos; gambling on video lottery/poker terminals; wagering on the Internet; and placing bets with a bookmaker, recently mostly via the Internet or smartphones [14]. Our results show that playing cards, dares and challenges or skills represent the most prevalent adolescent gambling activities (Table 3), while 6.6% (*N* = 24) of them bet regularly in sports betting houses and 5.8% (*N* = 21) do it online, 3% (*N* = 11) regularly bet on casino slot machines and 3.6% (*N* = 13) bet on live casino games, and 6.9% (*N* = 25) bet on casino games online.

**Table 3:** T-test for age differences

Variable	Age	N	Mean	SD	t
Psychological consequences of gambling	Younger	197	4.79	2.25	−2.65**
	Older	169	5.56	3.23	
Social consequences of gambling	Younger	197	4.27	1.15	−1.33
	Older	169	4.45	1.37	
Financial consequences of gambling	Younger	197	4.13	.83	−1.24
	Older	169	4.24	.88	
Family relations quality	Younger	197	7.55	6.18	1.12
	Older	169	6.81	6.48	
Problem gambling	Younger	197	8.79	2.05	−2.99**
	Older	169	9.72	3.74	
Risk behaviour	Younger	197	9.93	3.98	−4.70***
	Older	169	11.91	4.05	

Note: *p < .05; **p < .01; ***p < .001

Williams [37] has reported that gambling tendencies differed from study to study and location to location, as his study has shown that games of skill were the most popular gambling activity (51%), followed by card games (47%), sports betting (27%) and dice games (24%) [37]. This is not similar to some studies reporting greater rates of lottery participation by adolescents [38–40]. On the other hand, our present results are consistent with other studies in Alberta which found that adolescents favoured card games, games of skills, and sports betting as compared to other gambling activities [37, 41, 42]. Even though 23% of the Alberta sample reported one or more symptoms of problem gambling, only 2.5% met actual criteria for problem gambling [37]. This prevalence rate is lower than found in most other studies [40, 43–48]. It is also lower than the Shaffer et al. meta-analysis which reported prevalence rates of adolescent problem gambling to be 3.9% [49].

### The role of sociodemographic traits in adolescent problem gambling

Our first hypothesis **(H1)** assumed that sociodemographic traits have a significant impact on adolescent gambling. Our results show distinct gender differences (Table 2), with males reporting significantly higher gambling consequences on all scales (*p* < .001), but risk behaviour, as well (*p* < .05). Previous research established a relationship between male gender and gambling or problem gambling [42, 44, 46, 48, 50–53]. Sheela et al. found gender was significantly associated with adolescents’ gambling behaviour, with males having nearly three times the odds of being gamblers compared to girls [54]. A study by Di Nicola et al. has shown higher prevalence in gambling frequency among males, and significant gender differences in maladaptive gambling behaviour, with males scoring higher on the SOGS–RA score (*South Oaks Gambling Screen: Revised for adolescents*) [55]. Similarly, Elton-Marshall, Leatherdale and Turner have shown that online and land-based gambling was significantly more popular among males [56]. Mutti-Packer et al. indicated that gender was a significant predictor of baseline levels of gambling, as females reported lower initial levels of gambling problems [57], while studies by Williams [37] and Gonzalez-Roz et al. [58] showed no statistically significant differences between gender and gambling behaviour. However, a recent meta-analysis reported male gender to be among the strongest risk factors for problem gambling [12], which was confirmed in our present study results.

**Table 4:** ANOVA for differences in school success

Variable	School success	N	Mean	SD	Min	Max	F
Psychological consequences	Lower	54	6.24* > 2.3	3.46	4.00	18.00	6.14**
	Medium	194	5.13	2.81	4.00	20.00	
	Higher	118	4.67	2.16	4.00	19.00	
Social consequences	Lower	54	4.74	1.79	4.00	13.00	3.02
	Medium	194	4.28	1.04	4.00	12.00	
	Higher	118	4.31	1.28	4.00	14.00	
Financial consequences	Lower	54	4.26	.73	4.00	7.00	.71
	Medium	194	4.21	1.00	4.00	12.00	
	Higher	118	4.11	.61	4.00	10.00	
Family relations quality	Lower	54	5.19	6.48	.00	15.00	9.95***
	Medium	194	8.55* > 1.3	6.04	.00	15.00	
	Higher	118	5.94	6.24	.00	15.00	
Problem gambling	Lower	54	9.78	3.59	8.00	30.00	1.37
	Medium	194	9.23	3.03	8.00	25.00	
	Higher	118	8.97	2.57	8.00	21.00	
Risk behaviour	Lower	54	12.17	4.36	6.00	23.00	8.77***
	Medium	194	11.19	3.91	6.00	23.00	
	Higher	118	9.65* < 1.2	4.10	6.00	31.00	

Note: *p < .05; **p < .01; ***p < .001; Mean*: significant differences between groups 1,2,3

**Table 5:** T-test for differences in school type

Variable	School type	N	Mean	SD	t
Psychological consequences of gambling	Gymnasium	144	4.82	2.50	−1.82
	Vocational school	222	5.36	2.92	
Social consequences of gambling	Gymnasium	144	4.35	1.45	−.01
	Vocational school	222	4.36	1.12	
Financial consequences of gambling	Gymnasium	144	4.09	.45	−1.55
	Vocational school	222	4.24	1.03	
Family relations quality	Gymnasium	144	5.14	5.92	−5.22***
	Vocational school	222	8.55	6.22	
Problem gambling	Gymnasium	144	8.96	2.84	−1.37
	Vocational school	222	9.39	3.07	
Risk behaviour	Gymnasium	144	10.25	4.37	−2.22*
	Vocational school	222	11.23	3.92	

Note: *p < .05; **p < .01; ***p < .001

**Table 6:** Correlation matrix analysis

Variable		1	2	3	4	5	6	7	8	9	10	11
1 PSYCH CONSEQ GAMBLING	r	–	.58^***^	.28*^**^	.16^**^	.67*^**^	.27*^**^	.20^***^	−.35^***^	−.01	−.02	.21^***^
	N	366	366	366	366	366	366	366	366	366	366	366
2 SOCIAL CONSEQ GAMBLING	r	.58^***^	–	.39^***^	−.01	.72^***^	.22^***^	.13^*^	−.21^***^	.02	−.04	.14^**^
	N	366	366	366	366	366	366	366	366	366	366	366
3 FINANC CONSEQ GAMBL	r	.28^***^	.39^***^	–	.028	.66^***^	.17^**^	.091	−.20^***^	.05	.02	.06
	N	366	366	366	366	366	366	366	366	366	366	366
4 FAMILY RELATION QUALITY	r	,.6^**^	−.01	.03	–	.06	.49^***^	−.01	−.02	−.02	−.17^**^	−.01
	N	366	366	366	366	366	366	366	366	366	366	366
5 PROBLEM GAMBLING	r	.67^***^	.72^***^	.66^***^	.06	–	.26^***^	.19^***^	−.35^***^	.04	.000	.08
	N	366	366	366	366	366	366	366	366	366	366	366
6 RISK BEHAVIOUR	r	.27^***^	.22^***^	.17^**^	.49^***^	.26^***^	–	.16^**^	−.12^*^	.08	−.12^*^	.16^**^
	N	366	366	366	366	366	366	366	366	366	366	366
7 AGE	r	.20^***^	.13^*^	.09	−.01	.19^***^	.16^**^	–	−.14^**^	.06	−.05	.15^**^
	N	366	366	366	366	366	366	366	366	366	366	366
8 GENDER	r	−.35^***^	−.21^***^	−.20^***^	−.02	−.35^***^	−.12^*^	−.14^**^	–	.06	.05	−.16^**^
	N	366	366	366	366	366	366	366	366	366	366	366
9 FATHER EMPLOYMENT	r	−.01	.02	.05	−.02	.04	.08	.06	.06	–	.13^*^	.11^*^
	N	366	366	366	366	366	366	366	366	366	366	366
10 MOTHER EMPLOYMENT	r	−.02	−.04	.02	−.17^**^	.01	−.12^*^	−.05	.05	.13^*^	–	.01
	N	366	366	366	366	366	366	366	366	366	366	366
11 SCHOOL SUCCESS	r	.20^***^	.14^**^	.06	−.01	.08	.16^**^	.15^**^	−.16^**^	.11^*^	.01	–
	N	366	366	366	366	366	366	366	366	366	366	366

Note: *p*<.05*; *p*<.01**; *p*<.001***

Only significant coefficients equal to or higher than 0.5 were highlighted in bold. Also, significant coefficients with only negative significant prefixes for a certain variable (gender) were presented in bold, as it shows a trend in correlation

**Table 7:** Regression analysis for Problem gambling (*N* = 366)

Problem gambling	Model 1 (Soc.-demo. characteristics)			Model 2 (Risk-protective factors)		
	*B*	*SE(B)*	*β*	*B*	*SE(B)*	*β*
Age	.64	.27	.34*	−.06	.14	−.03
Gender	−2.11	.31	−.34***	−.55	.17	−.09**
Father’s education	−.36	.17	−.11*	−.12	.09	−.04
Mother’s education	.15	.17	.05	−.04	.09	−.01
Father’s employment	.07	.09	.04	.03	.05	.02
Mother’s employment	.03	.11	.01	.02	.06	.01
Grade / class	−1.19	.86	−.19	.45	.45	.08
School success	−.08	.19	−.02	−.34	.10	−.09**
Psychol. conseq. of gambling				.34	.04	.32***
Social conseq. of gambling				.87	.08	.37***
Financial conseq. of gambling				1.39	.09	.39***
Family relations quality				−0.02	.01	−.01
Risk behaviour				.04	.02	.05
***R***^***2***^	**.160**			**.781**		
R Square Change	**.160**			**.621**		
Adjusted ***R***^***2***^	**.141**			**.773**		
df	**8, 357**			**13, 352**		
***F***	8.482***			96.559***		
***F*** **for change in** ***R***^***2***^	8.48***			199.71***		

Note: *p*<.05*; *p*<.01**; *p*<.001***

In their investigation on the role of age, Sheela et al. did not find significant associations between age and adolescent gambling behaviour [54], but an earlier age of first gambling activity was established as a risk factor [59]. Several research studies found no significant age differences in psychological consequences of gambling, social consequences of gambling, financial consequences of gambling and problem gambling behaviour, as well [30, 55], while McBride and Derevensky showed that a greater proportion of non-gamblers were under the age of 18 years, whereas a significantly greater proportion of social gamblers were aged 21–24 [60]. In a study conducted by Kristiansen and Jensen, the proportions of *at-risk* gamblers and problem gamblers were significantly higher among older age groups [61]. Kristiansen and Jensen reported a weak, non-significant relationship between age and the SOGS-RA score, with older respondents reporting more gambling problems [61]. This is consistent with our findings and could imply that gambling behaviour is related to personal maturation, more agency and social emancipation, but also indicate that preventive actions need to be targeted at specific age groups. As evidence indicates, wagering something of value on an uncertain event often begins as early as grade school [44], with age 11 being the average age of onset found in a couple of major studies [25, 47]. Still, our results show a worrying age decline as the earliest reported age of gambling initiation was 7, while it was most prevalent at ages 14, 15 and 18, implying a need for an earlier onset and optimization of efficient prevention programs in schools and community activities.

### Family life, parental traits and school related factors in problem gambling

The second hypothesis **(H2)** assumed that family life satisfaction, parental traits and school related factors represent protective factors in gambling aetiology. Our research has shown (Table 4) that lower school achievers report significantly higher psychological consequences of gambling in relation to better students (*p* < .01). A large amount of international research has found that problem gamblers tend to be concentrated among those lacking college education, and who have dropped out of high school, while several studies have demonstrated correlations between higher spending on gambling and lower levels of education [62]. Gambling during adolescence has been linked with psychiatric, social, and substance misuse problems in adulthood [63]. Both recreational and problem gambling have been associated with adverse social functioning and mental health in adolescence including poor school performance and difficulties with aggression and mood [53, 64, 65]. Foster et al. also reported that students who gambled on school grounds had poorer academic performance [66], while a study by Gonzalez-Roz et al. showed no statistically significant differences between school success and gambling behaviour [58]. Our results also demonstrate significantly higher risk behaviour activity (*p* < .001) and significantly lower family life satisfaction among participants who report the lowest school achievement (*p* < .001), so the intensity of psychological consequences could also stem and be related to a feeling of rejection due to problems in family, school failure and risk behaviour participation.

Our results showed no significant differences in gambling behaviour or its consequences in relation to the school type participants attended, but indicated significantly higher risk behaviour for vocational school students. These results are in line with similar studies which have shown that vocational school students have significantly higher risk behaviour prevalence than gymnasium students, with no significant school type differences in gambling behaviour [53, 67].

The place of residence (urban/rural) was significant for the quality of family relations, with rural participants reporting both significantly higher family life satisfaction, but more risk behaviour as well. Family structure (both parents/other) was significant for both family life quality and risk behaviour, with participants from structurally deficient families reporting higher family life satisfaction and riskier behaviour. Similarly, Foster et al. also did not establish significant gambling differences in relation to family structure [66], but Canale et al. reported that two family characteristics increased adolescent gambling – living with unrelated others or a single parent, and in poor families [68]. Our results show the mothers’ employment status was significant only for family life satisfaction, with participants who had employed mothers reporting more satisfaction. The fathers’ employment status was not significant for any of the examined variables. Both the mothers and fathers’ educational level did not show significance in relation to any of the examined variables. Even though our study did not investigate parental gambling behaviours, previous research has positively correlated parental gambling and adolescent gambling, with children of problem gamblers tending to gamble earlier than their peers [10, 44, 52, 69, 70], implying the need to educate parents on family risk factors for problem gambling.

### The consequences of adolescent problem gambling

The third hypothesis **(H3)** assumed gambling had significant psychological, social and financial consequences on adolescents. Our results established a positive correlation between the psychological, financial and social consequences with problem gambling (Table 6), consistent with previous research on emotional problems and gambling, for example a positive relation between time spent gambling and depression [52, 71], also found in a study by Williams [37] and Rossen et al. [72], who reported that students with unhealthy gambling practices reported significantly more mental health issues and other addictions/risky behaviours [72]. Among the high-risk behaviours, adolescents that smoke, consume alcohol and participate in physical fights had significantly higher odds for gambling addiction. In a study by Castrén et al. both smoking and drinking for intoxication were significantly associated with *at-risk* and problem gambling compared with non-smokers and respondents who had not been drinking for intoxication [73]. Gambling frequency has been found to be highly associated with other forms of antisocial activity, for example, delinquency [74]. Additionally, Williams [37] points to a number of studies that reported a positive relationship between risk-taking and gambling, which was confirmed by a meta-analysis by Dowling et al. [12].

### Predictors of adolescent problem gambling

Our hierarchical regression analysis results show that age, gender, school success and the father’s education level all significantly predicted problem gambling in adolescents, with older male adolescents who struggle academically and have lower educated fathers being at greater risk (Table 7). Our results also established that more intense psychological, social and financial consequences in gambling positively predicted problem gambling. For example, Gupta and Derevensky [9] reported that excessive gambling in boys caused emotion-focused coping strategies, such as anger, frustration or anxiety during negative events. Similarly, Williams found a positive relationship between time spent gambling and depression [37]. All our regression results are in line with Rossen et al., who found that males are disproportionately at risk of problem gambling [72]. The same study found that students with unhealthy gambling practices were significantly more likely to report co-existing mental health issues (e.g. depression and suicide attempts) and other addictions/risky behaviours (e.g. use of alcohol and weekly cigarette smoking). For example, Williams found that time spent gambling was a significant predictor for higher levels of impulsivity, possessing more positive attitudes towards gambling, having been in trouble with the police, having suffered from depression, possessing less knowledge about gambling and having greater cognitive errors [37]. While Ste-Marie, Gupta and Derevensky found that gamblers with the highest scores on state and trait anxiety, as well as for social stress, were likely to meet the criteria for probable pathological gambling [28], a recent meta-analysis on problem gambling revealed that aggression, anxiety symptoms, attention problems, a big early gambling win, dispositional attention, psychological distress (including internalising symptoms), religious attendance and suicidal ideation were not significantly associated with subsequent problem gambling, so results are inconclusive [12]. Even though our results did not show a significant role of family structure in gambling, a study conducted by Allami et al. has shown that, at age of 16, parent–child connectedness and higher parental involvement significantly predicted fewer gambling problems, while peer connectedness may have an effect on problem gambling, but that effect likely depends on peer gambling behaviour [75]. A recent meta-analysis on gambling established parent supervision and socio-economic status as significantly negatively associated with subsequent problem gambling [12]. Our regression analysis did not establish family life quality or parent employment as significant predictors, while Jackson et al. found that participating in gambling activities was associated with parental employment [76]. They found no significant associations between gambling and family structure, nor between gambling and parental education. The same study also found a positive association between gambling involvement and depressive symptomology, deliberate self-harm and arguments with others; as it did between gambling participation and engagement in substance use and antisocial behaviours. These behaviours, with the exception of smoking, were significant predictors of greater involvement in gambling [76]. Interestingly, a study by Williams established a positive attitude toward gambling as the most consistent predictor of gambling behaviour, as well as problem gambling [37]. Among other established predictors, Williams found that larger amounts of money won while gambling, positive attitudes towards gambling, impulsivity, more gambling-related cognitive errors, greater risk-taking and less knowledge about gambling, were the variables that significantly contributed to the prediction of higher gambling frequency in order of predictability [37]. Similarly, having won a large sum of money gambling was the best predictor of increased time spent gambling. In addition, spending more time gambling was also associated with higher levels of impulsivity, possessing more positive attitudes towards gambling, having been in trouble with the police, having suffered from depression, possessing less knowledge about gambling and having greater cognitive errors, in descending order of predictability [37]. Reductions in positive attitudes towards gambling, prior trouble with the police and increased knowledge were the only three variables that significantly predicted decreased gambling frequency [37]. Surprisingly, even though we did not establish predictive relations between risky behaviour and problem gambling, it has been found to be highly associated with other forms of antisocial activity. For example, delinquency has been found to be positively related to both gambling frequency and problem gambling [73].

## Implications for research and practice

Problem gambling is a complex research area, so findings are sometimes contradictory even though such behaviour is proven to disrupt personal, family, financial, professional and social relations [62]. Our study was focused on explicating the role of sociodemographic traits, family relations quality and risk behaviour in adolescent problem gambling. Such quantitative studies on sociodemographic factors related to personal characteristics, family life satisfaction and school surroundings are relatively scarce. While many adolescents gamble occasionally and don’t experience significant problems, studies suggest they constitute a vulnerable population for gambling problems, especially with the onset of online gambling. Low numbers of treatment seeking adolescent problem gamblers may be related to the fact that they do not consider disruptive gambling as problematic and underestimate its impact. Treatment-wise, high comorbidity with other disorders may obscure problem gambling, as other problems get more clinical attention, while the heterogeneity within a relatively narrow age range implies the need for divesified approaches [77]. Our results indicate *3–7%* of adolescents who regularly participate in serious gambling activities, in line with a study by Volberg et al., who reported 2–8% of adolescents having serious gambling problems, with another 10–15% being at-risk for the development of a gambling problem, especially young adults ages 18–25 [78]. Our regression analysis showed that the average at-risk individuals are older male adolescents who struggle academically, have lower educated fathers, attend vocational schools and report low family life satisfaction. It is interesting we established that problem gambling was positively predicted by psychological, social and financial consequences, in accordance with studies that established high comorbidity of problem gambling with psychological conditions [79], but their relation remains unclear as to the nature or relationship of the three variables in the prevention or inhibition of gambling. Adolescent problem gamblers use less task-focused coping and more avoidance coping strategies than non-gamblers, while male excessive gamblers demonstrate more emotion-focused coping strategies, such as anger, frustration or anxiety during negative events [80]. Therefore, gambling behaviour is closely related to feelings of serious social, psychological and financial consequences for adolescents. Similar to adults with a gambling disorder, adolescents report having a preoccupation with gambling; repeated attempts at recouping losses; increasing wagers to reach a physiological level of excitement; lying to others about gambling; with anxiety and depression when trying to reduce their gambling. A considerable number of adolescents report gambling as a coping mechanism to psychologically escape daily problems (parental, peer, and school-related) and other mental health issues [13, 81]. Surprisingly, family life quality, parent employment and risky behaviour were not established as significant predictors for problem gambling in our study, so the role of parents and family should be further investigated. Lussier et al. examined the concept of resilience for youth gambling problems and other adolescent high-risk behaviours and suggested that family cohesion was an important element in adolescent resilience [17]. Exposure to an object of addiction at a young age or exposure to a parent’s addiction could both increase the likelihood of developing an addiction. One recent study found that children of pathological gamblers were four times more likely to develop the disorder, so it is important that health professionals, teachers and other experts understand the family background [82]. Recent findings suggest that some of the early factors associated with the onset of problem gambling in cross-sectional studies have not been identified in subsequent longitudinal studies, which suggests that these factors may be, in fact, consequences of problem gambling or co-exist because they share common causes [12]. Therefore, longitudinal studies shift the policy focus from elements that co-occur with problem gambling in youth at a certain cross-sectional point in time to predictive factors of gambling at a future time-point, including adulthood. Adolescent gambling behaviour should be viewed on a continuum, from non-gambling to social or occasional and recreational gambling to at-risk gambling, up to problem or pathological, compulsive or disordered gambling [14]. In conclusion, clinical and research evidence suggests that efficient prevention strategies have an impact through providing facts about gambling which improve knowledge and significantly reduce misconceptions, resulting in more realistic attitudes towards gambling [83, 84]. Still, their effectiveness needs further investigation, thus emphasising the need for future efficient preventive social and educational policy.

### Limitations

Several limitations of the current study should be noted. The research sample was small; therefore, conclusions of a larger scale and results generalization are out of the scope of this study. In addition, the extent of underreporting or over-reporting of behaviours cannot be determined, although the survey questions demonstrate good intercorrelational reliability. The “Gambling social consequences” scale demonstrated lower reliability, even though standardised survey instruments were implemented. Sociodemographic traits, family relations, risk behaviour and problem gambling experiences were self-reported, but previous studies have shown these measures to be valid [85, 86]. Despite these limitations, a strength of the study was the use of instruments with reliable psychometric properties to measure adolescent gambling behaviour and its social, psychological and financial consequences.

## Conclusions

The results of our study indicate that there is a distinct role of socio-demographic characteristics in the aetiology of adolescent problem gambling, that are mostly related to gender, age, academic achievement and the father’s educational level. What begins as an exciting benign form of entertainment for most, could result in serious problems for an identifiable group of young people. It should be noticed that our findings clearly indicate an important relation between adolescent gambling behaviour and very serious psychological, social and financial consequences, as well as risk behaviour. There is a constellation of factors that likely place certain individuals at high risk for problem gambling. The increasing awareness that the aetiology underlying gambling problems is not universal, that risk factors may be different for individuals, and that there are a number of distinct pathways which could lead to pathological or disordered gambling, pose new important questions for researchers and practitioners on the structure and focus of preventive activities.

## Data Availability

All research data is available upon demand, and was submitted to the Editorial Office in the publication process. Requests for data and materials should be addressed to the corresponding author.
